# Speed of Human Biological Form and Motion Processing

**DOI:** 10.1371/journal.pone.0069396

**Published:** 2013-07-24

**Authors:** George Buzzell, Laura Chubb, Ashley S. Safford, James C. Thompson, Craig G. McDonald

**Affiliations:** Department of Psychology, George Mason University, Fairfax, Virginia, United States of America; University of Muenster, Germany

## Abstract

Recent work suggests that biological motion processing can begin within ~110 ms of stimulus onset, as indexed by the P1 component of the event-related potential (ERP). Here, we investigated whether modulation of the P1 component reflects configural processing alone, rather than the processing of both configuration and motion cues. A three-stimulus oddball task was employed to evaluate bottom-up processing of biological motion. Intact point-light walkers (PLWs) or scrambled PLWs served as distractor stimuli, whereas point-light displays of tool motion served as standard and target stimuli. In a second experiment, the same design was used, but the dynamic stimuli were replaced with static point-light displays. The first experiment revealed that dynamic PLWs elicited a larger P1 as compared to scrambled PLWs. A similar P1 increase was also observed for static PLWs in the second experiment, indicating that these stimuli were more salient than static, scrambled PLWs. These findings suggest that the visual system can rapidly extract global form information from static PLWs and that the observed P1 effect for dynamic PLWs is not dependent on the presence of motion cues. Finally, we found that the N1 component was sensitive to dynamic, but not static, PLWs, suggesting that this component reflects the processing of both form and motion information. The sensitivity of P1 to static PLWs has implications for dynamic form models of biological motion processing that posit temporal integration of configural cues present in individual frames of PLW animations.

## Introduction

Humans are able to perceive the actions of others with relative ease, even when their movements are reduced only to points of light [1]. Although much work has been done to determine the anatomical regions that give rise to this remarkable process (for a review, see 2) the underlying mechanism remains unclear. Recent neuroimaging evidence suggests that coherent biological motion perception requires the integration of local motion and configural cues [3]. However, neuropsychological and modeling studies suggest that this phenomenon can be explained in terms of configural processing alone [4–6]. A better understanding of the time course of configural and motion cue processing can be expected to help resolve competing views of biological motion perception.

There is currently no consensus on the temporal dynamics of form and motion cue processing. There is some evidence from event-related potential (ERP) studies that the processing of human biological motion begins in the latency range of the occipital-temporal N1 component [7,8], which peaks approximately 170-210 ms following stimulus onset. Such activity has been suggested to reflect the integration of form and motion information, allowing for the generation of coherent biological motion percepts [9]. However, other studies have shown that human motion can elicit a selective response prior to the peak of the N1 component [10] and even as early as the P1 component [11], which occurs ~110 ms following stimulus onset. Such a P1 modulation is consistent with MEG work by *Pavlova* and colleagues [12,13] showing increased parietal gamma responses at approximately 120-130 ms after stimulus onset.

The results of Krakowski et al. [11] showed that the P1 component was sensitive to biological motion regardless of whether attention was directed toward the global configuration of the point-light walker (PLW) stimuli or not. This suggests that early processing of biological motion, as indexed by P1 modulation, occurs in a reflexive, bottom-up fashion. This finding is in accord with behavioral evidence that biological motion processing can occur via low-level visual mechanisms that are not dependent on top-down attentional control [14] (but see 15).

It is possible that differences in ERP quantification may account for the failure of some previous investigations [7,8] to detect differences during the P1 time range. Nonetheless, it remains unclear whether modulation of the P1 component is indicative of the processing of both form and motion cues. Because intact PLWs preserve both global motion and configural information [1], this early effect could reflect configural processing alone.

In an effort to determine whether neural activity in the P1 time range reflects the processing of both form and motion, we conducted two experiments which differed only with respect to whether point-light stimuli were dynamic (containing both form and motion information) or static (containing only form information). In order to directly study bottom-up processing of biological motion stimuli, we chose to utilize a three-stimulus oddball task. This task has frequently been used to evaluate reflexive processing of task-irrelevant distractor stimuli [16]. In our version of the task, intact or scrambled PLWs served as distractors; these stimuli were embedded in a stimulus train of point-light tool displays serving as standard and target stimuli. Given that the PLWs were task-irrelevant (i.e., to be ignored) and infrequently presented (i.e., unexpected), early sensory processing of these stimuli can be expected to occur in a reflexive, bottom-up manner, without endogenous attentional influences. In the first experiment the stimuli were dynamic, whereas in the second experiment the stimuli were static.

Based on the findings of Krakowski et al. [11], we predicted that dynamic, intact PLWs would elicit an increase in the occipital P1, followed by an increase in the occipital-temporal N1 component. The use of static PLWs in the second experiment allowed us to explore whether any effects observed in experiment one reflect the processing of both configuration and motion cues, as opposed to configuration cues alone. A similar P1 effect in both experiments one and two could be taken as evidence that such an effect is not dependent on the presence of local motion cues. Alternatively, modulation of the P1 only in experiment one would provide evidence that this component reflects the processing of both form and motion information.

## Materials and Methods

### Participants

Participants were recruited from the George Mason University undergraduate population and the surrounding community (19 participants for experiment one, 15 participants for experiment two). Healthy young male and female adults took part in the study, all of whom had self-reported normal (or corrected to normal) vision and no known neurological deficits. Participants recruited from the undergraduate population were provided course credit for participation, while those recruited from the surrounding community were provided with nominal compensation for their time. All participants provided written informed consent after having been explained the procedures of the study. All procedures were approved by the Office of Research Subject Protections at George Mason University.

### Stimuli

**Figure 1:**
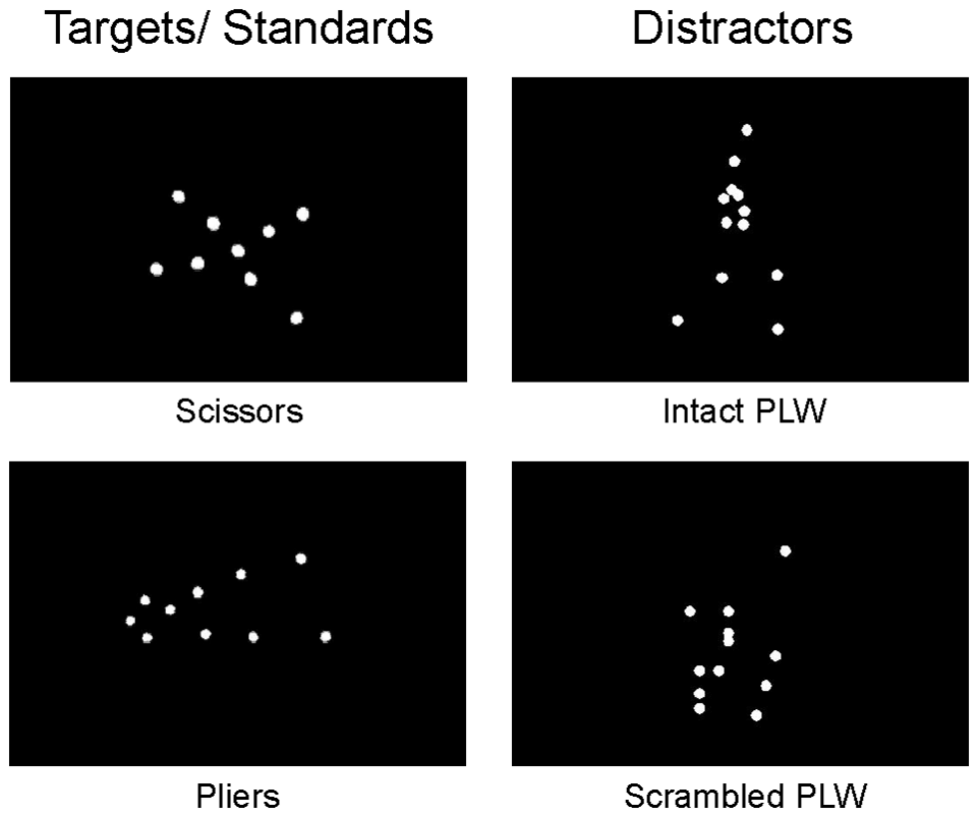
Experimental Stimuli. Static representations of the four stimulus types used in experiments one and two. In experiment one, participants discriminated between point-light animations of tool motion (scissors and pliers) while either intact or scrambled PLW stimuli were infrequently presented as distractor stimuli. Randomly selected static frames from the animations used in experiment one were used as stimuli for experiment two.

Nine different point-light animations (comprised of 12 white dots on a black background) depicting a human facing in a rightward direction and appearing to walk in place were used for experiment one. Each PLW animation depicted one full gait cycle and was presented at 40 frames/sec for a total presentation time of one second. For scrambled PLWs, local motion was applied to 12 white dots at locations drawn from a two-dimensional normal distribution with mean (and standard deviation) location determined from the mean (and standard deviation) location across joints of the intact PLW. This scrambling procedure ensured that the retinal displacement of the intact and scrambled PLWs was comparable. Point-light animations depicting the typical motion of scissors and pliers (“tool motion”) were presented at a reduced frame rate of 29 frames/second. Detailed methods describing the construction of the tool and PLW stimuli can be found in [9,17]. For experiment two, the PLW stimuli consisted of nine randomly selected frames from the animations used in experiment one (presented for one second). Tool point-light displays consisted of randomly selected frames of the tool animations. See Figure 1 for static representations of the four stimulus types used. All stimuli were preceded by a green fixation square, displayed for 500 ms and were followed by a red fixation square presented for a variable duration between 500 and 1000ms (randomly drawn from a uniform distribution). Stimuli were displayed using Presentation software (Neurobehavioral Systems, California, USA).

### Procedure

After first practicing the task, participants completed eight blocks (140 trials each) of a visual three-stimulus oddball task involving centrally presented stimuli. Participants were required to press the left mouse button in response to infrequently (*P* = .15) presented target point-light tool motion and withhold responses to both frequently (*P* = .70) presented standard point-light tool motion and infrequently (*P* = .15) presented distractor intact or scrambled PLWs. The type of tool motion (pliers or scissors) serving as targets and standards, as well as the type of PLW (scrambled or intact) serving as the infrequently presented distractor, were counterbalanced within subjects across the eight blocks. Participants were encouraged to take breaks between blocks in order to reduce fatigue.

### EEG Data Collection and Processing

EEG data was collected using a Compumedics EEG system and SCAN 4.3 data acquisition software (Compumedics, North Carolina, USA). Data was sampled at 500 hz and recorded with an online band-pass filter with cutoffs at .1 and 70 Hz. Ag/AgCl electrode locations followed the standard 10-20 system arrangement and were embedded within an electrode cap. Data was recorded from the following electrode locations: Fp1, F7, F3, FT7, FC3, T7, C3, TP7, CP3, P7, P3, O1, Fz, Cz, Pz, Oz, FP2, F8, F4, FT8, FC4, T8, C4, TP8, CP4, P8, P4, O2. Data was recorded using an in-cap ground (located between FPz and Fz) and reference electrode (located between Cz and CPz). In order to monitor for blinks and eye movements, electrooculogram activity was recorded using two sets of bipolar electrode montages, located at the outer canthus of each eye, as well as above and below the left eye.

Following acquisition, all EEG data was processed using the EEGLAB toolbox [18] and ERPLAB plug-in (University of California, California, USA) designed to run in the MATLAB programming environment (MathWorks, Massachusetts, USA). The EEG data of each subject was filtered using a Butterworth filter between .1 and 30 Hz. Data was then re-referenced to the average of all electrode locations. For each event of interest, data was epoched to 200 ms before and 800 ms after the stimulus onset. In order to remove ocular artifacts, a rejection criterion of +/- 75 µV was set for both the vertical and horizontal ocular electrode montages. Each participant’s data was then manually inspected to remove further contamination by EOG or EMG activity. After removing participants that had too few trials following artifact rejection (less than 34 trials for each stimulus), 12 participants remained in each experiment for ERP analysis.

### ERP Component Identification and Analysis

Mean amplitude analysis windows were identified in a three-step process. First, a grand average waveform was constructed for epochs time-locked to the onset of both the intact and scrambled PLW stimuli. For each component of interest, the grand average waveform was examined at a subset of electrode locations. Consistent with previous work, we selected the three occipital electrodes, O1, O2 and Oz, for evaluation of the P1 component [19,20]. For the N1 component, we selected the P7 and P8 electrodes based on a number of previous studies that have characterized this component at or near these electrode sites [7,9,10]. A larger subset of electrodes (P3, P4, Pz, C3, C4, Cz, F3, F4 and Fz) was used to evaluate the P3a as the topography of this component has been found to be inconsistent across investigations [16,21,22]. After identifying the electrode location where amplitude was maximal, a predefined window (20 ms for P1, 32 ms for N1 and 40 ms for P3a) centered on the peak amplitude was used to calculate mean amplitude at all electrodes in a given set.

Following the identification of analysis windows, a series of 2 factor (stimulus type by electrode location) ANOVAS were carried out for each component. Where appropriate, a Greenhouse-Geisser epsilon adjustment was used to correct for violations of sphericity (only raw degrees of freedom are reported below). The chance of a type one error during follow-up comparisons was controlled with the use of a Bonferroni correction for multiple comparisons.

## Results

### Experiment One

#### Behavioral Data

The mean hit rate and reaction time for targets was 98.36% (SD = 2.45%) and 620 ms (SD = 56 ms), respectively. The mean false positive rate was .48% (SD = .25%) for standards and .25% (SD = .47%) for distractors.

#### ERP Data

**Figure 2:**
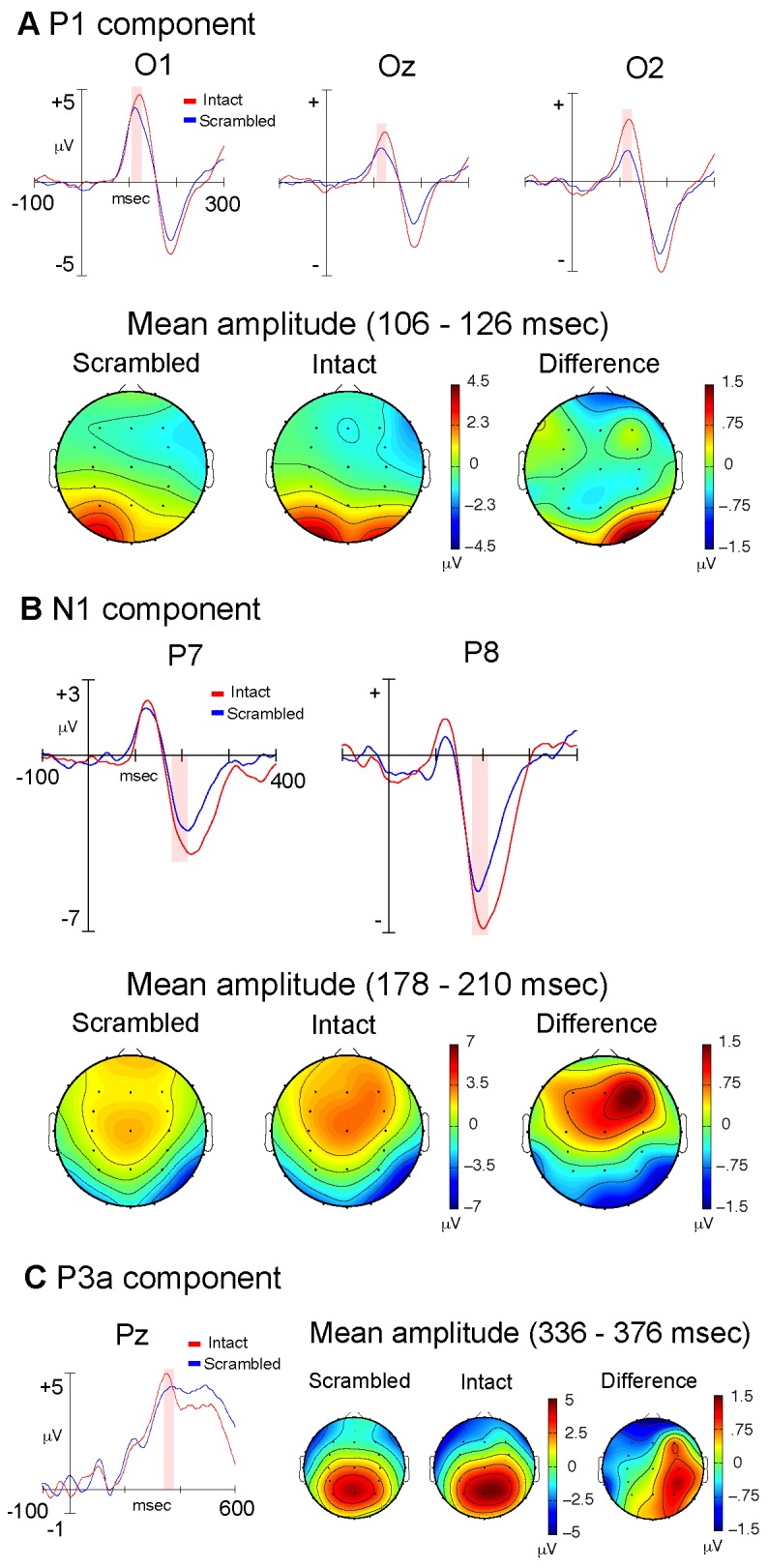
Experiment One: Dynamic Stimuli. Stimulus-locked, grand average ERP waveforms and topographic plots for the P1 (A), N1 (B) and P3a components (C), comparing intact (red) and scrambled (blue) dynamic PLW stimuli. The shaded areas shown in the waveform plots indicate the ERP measurement windows. The topographic plots illustrate the scalp distribution of mean component amplitudes derived using the indicated windows.

Analysis of the P1 component (106–126 ms) revealed a significant main effect of electrode location (F(2,10) = 5.97; p = .018) as well as a significant electrode by stimulus type interaction (F(2,10) = 6.32; p = .009). Post-hoc analyses (Bonferroni correction; alpha = .05/3 = .017) revealed that the interaction was due to a right lateralized (electrode O2) increase in amplitude elicited by intact PLWs (t(1,11) = 2.96; p = .013) (Figure 2a). Analysis of the N1 component (178–210 ms) resulted in a main effect of stimulus type (F(1,11) = 5.65; p = .037), with amplitude being greater in response to intact PLWs. In addition, a main effect of electrode location (F(1,11) = 14.19; p = .003) was identified, with amplitude being significantly greater for the right (P8) electrode location (Figure 2b). It should be noted that the fronto-central positivity present in the N1 topographic plots likely reflects the Vertex Positive Potential (VPP), the positive end of this component’s dipole [23]. Analysis of the P3a component (336–376 ms) resulted in a main effect of electrode location (F(8,4) = 13.67; p = .001). In order to further investigate this effect, lateral and midline electrodes were collapsed at the parietal, central and frontal scalp locations and a series of post-hoc t-tests (Bonferroni correction; alpha = .05/3 = .017) were carried out to evaluate differences in amplitude along the rostro-caudal axis. The analyses revealed a monotonic increase in amplitude, progressing from frontal to parietal regions of the scalp (all p< .017) (Figure 2c). We note here that, although the P3a typically has a more frontal topography (at least in the auditory modality) it has been observed to be maximal at more posterior sites in the visual modality [21,22].

### Experiment Two

#### Behavioral Data

The mean hit rate and reaction time for targets was 96.23% (SD = .05%) and 605 ms (SD = 71 ms), respectively. The mean false positive rates for standards and distractors were .73% (SD = .49%) and 1.54% (SD = 2.7%), respectively.

#### ERP Data

**Figure 3:**
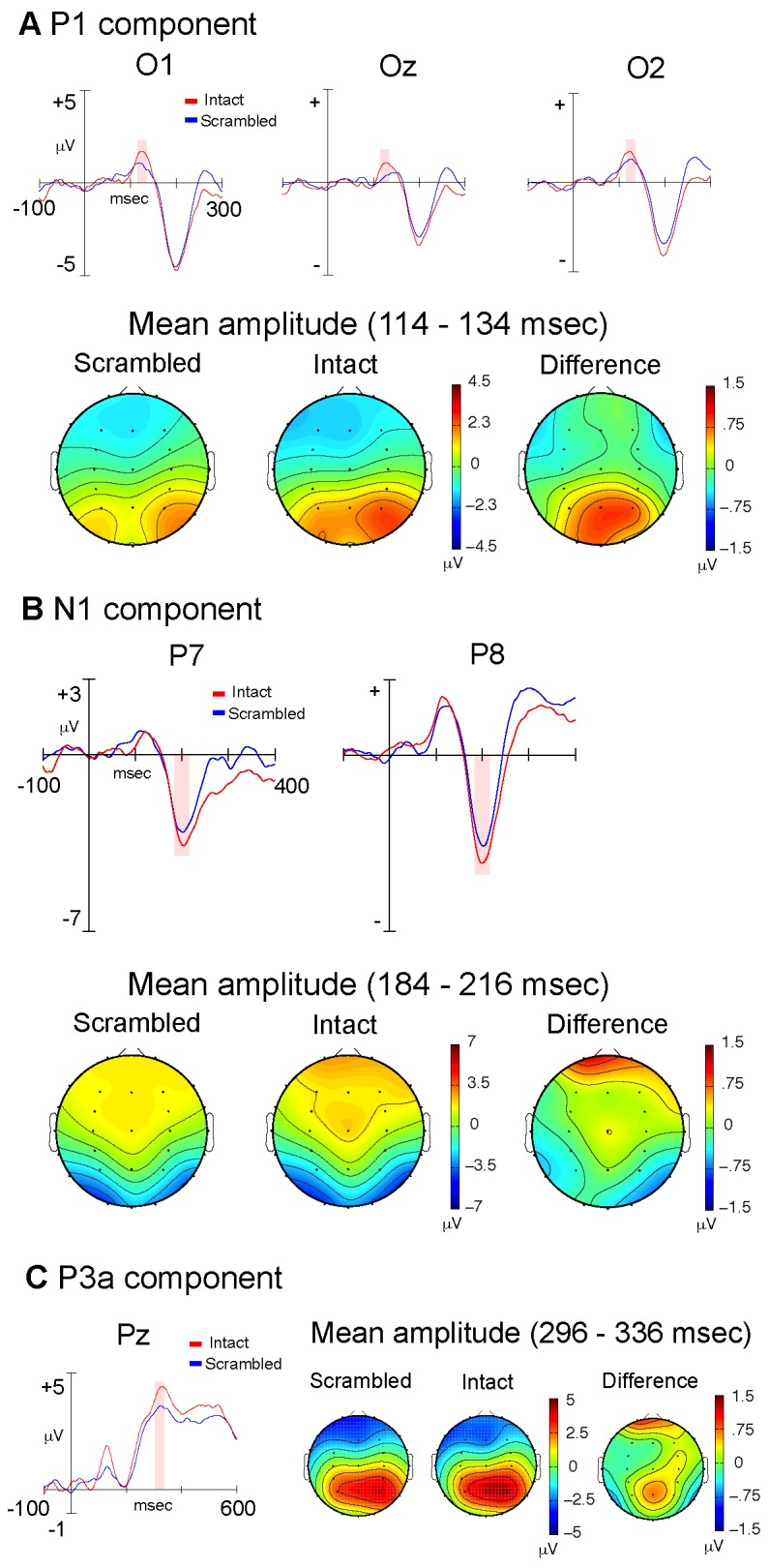
Experiment Two: Static Stimuli. Stimulus-locked, grand average ERP waveforms and topographic plots for the P1 (A), N1 (B) and P3a components (C), comparing intact (red) and scrambled (blue) static PLW stimuli. The shaded areas shown in the waveform plots indicate the ERP measurement windows. The topographic plots illustrate the scalp distribution of mean component amplitudes derived using the indicated windows.

Analysis of the P1 component (114–134 ms) revealed a main effect of stimulus type (F(1,11) = 5.23; p = .043), with increased amplitude in response to intact PLWs (Figure 3a). However, there was no significant stimulus by electrode interaction (F(2,10) = .42; p = .551). There were no significant effects with respect to the N1 component (184–216 ms); however there was a trend for a main effect of stimulus type (F(1,11) = 4.12; p = .067), with increased amplitude in response to intact PLWs (Figure 3b). Analysis of the P3a component (296–336 ms) resulted in a main effect of electrode location (F(8,4) = 40.14; p = .001). A series of post-hoc analyses, following the same procedure as that used in experiment 1, revealed a monotonic increase in amplitude, progressing from frontal to parietal regions of the scalp (all p< .017) (Figure 3c).

## Discussion

The primary finding of the present study was that, under experimental conditions designed to evaluate bottom-up processing, the P1 component of the ERP was sensitive to both static and dynamic PLWs. This finding suggests that the visual system can rapidly extract global form information from static PLW stimuli and that the sensitivity of P1 to dynamic PLWs is likely not a function of the presence of motion cues. The N1 effect observed for dynamic PLWs is in line with an increasing body of research showing similar results [7–11]. In addition, the lack of sensitivity of N1 to the static PLWs supports the view that this component indexes the integration of form and motion information [9].

One possible interpretation of our data might be that the intact PLWs produced greater reflexive attentional capture because they shared features with the target stimuli; both the intact PLWs and tool point-light displays contained meaningful configural information, whereas the scrambled PLWs did not. Therefore, it is possible that an attentional bias toward coherent configural information (necessary for target detection) resulted in a bias toward intact PLWs that was not present for scrambled PLWs. However, previous work using the three-stimulus oddball paradigm suggests that this is not the case. It has been consistently shown that the orienting response to distractors, as indexed by the P3a component, is stronger when the distractors are more dissimilar to the standards and targets. This phenomenon is even more pronounced when the target and standard stimuli are similar in appearance [16,24]. In the present study the standards and targets had a high degree of similarity (both were tools of similar appearance and movement dynamic), and their assignment as target and standard stimuli was counterbalanced across blocks. Thus, one could argue that the scrambled PLWs, which were the only stimuli without coherent configural information, would have been expected to elicit a stronger orienting reflex than the intact PLWs. Clearly, the opposite pattern of results was observed in the present study--the P1 elicited by intact PLWs was significantly larger than that elicited by scrambled PLWs. Moreover, the P3a elicited by these stimuli did not differ significantly (although it is possible that this could be due to the task not being difficult enough to elicit a robust P3a). We also note that mismatches between standard and ‘oddball’ stimuli appear to be first registered by the visual mismatch negativity (vMMN), a component with significantly longer latency than the P1 [25]. Thus, we argue that the observed P1 effects likely reflect the inherent properties of the intact and scrambled PLWs, and were not significantly affected by the context in which the stimuli were experienced. However, future work should be undertaken to definitively exclude the possibility that context may play a role in the observed results.

It might be argued that the observed modulation of P1 reflects the influence of top-down attention. However, this is unlikely given that we used a paradigm in which the intact and scrambled PLWs were task-irrelevant and unexpected. Consequently, these stimuli were subject to a bottom-up processing bias. We also note that the vast majority of studies showing an effect of top-down attention on P1 are studies of visuospatial attention [26]. Moreover, spatial attention does not appear to affect P1 when stimuli are presented at fixation [27]. In the present study, stimuli were centrally presented and spatial attention was not manipulated.

Feature-based, top-down attentional influences are also unlikely to have affected P1 in the present study, as such influences have only been observed when there is simultaneous competition between overlapping stimuli [28]. In addition, P1 does not appear to be sensitive to changes in endogenous attention in discrimination tasks in which stimuli are presented near fixation [29]. Given these considerations, it is unlikely that the observed sensitivity of P1 to PLWs can be attributed to top-down attention or the context in which the stimuli were experienced. Rather, the most parsimonious explanation is that the intact PLWs were more salient than the scrambled PLWs, resulting in greater bottom-up activation of visual cortex. We note that although the data suggest that the configuration of the stimuli played a role in modulating P1, they do not allow us to conclude that configural processing is complete at this early point in the processing stream.

Although the P1 component has previously been shown to be sensitive to faces [30] (but see 31), it has not previously been shown to be sensitive to static images of PLWs. In addition, it is commonly assumed that the individual frames comprising the dynamic PLW cannot be consciously recognized as human by naïve observers [1], which was also reflected by anecdotal evidence provided by informal conversations with the participants of experiment two. Nonetheless, the static PLWs produced an increase in neuronal activation as indexed by the P1 component. It is not clear whether this P1 effect reflects processing exclusive to the human form, as a similar effect might be expected for other globally coherent static images. Further research that directly compares the electrophysiological response to point-light walkers and other point light objects, such as tools, is needed to further clarify this result.

It is possible that differences in ERP quantification may explain why some previous investigations did not report differences with respect to the P1 component [7,8,10]. Indeed, it appears that Hirai et al. [7] did not evaluate the ERP in the time range of the P1 component. However, if one inspects the waveforms presented in this paper, it appears that there is a P1 enhancement to intact PLWs over occipitotemporal regions. Notably, in another study, the same group investigated the contributions of form and motion cues to biological motion processing and found a very early ERP difference (< 100 ms) that was attributed to differences in the spatial configuration of the PLW displays [8]. This difference manifested as a greater negativity prior to the P1 peak for scrambled stimuli, although it is possible that this effect was driven by differences in P1 amplitude. Specifically, it is possible that a greater negativity was observed because a diminished P1 component allowed for an early deflection of opposite polarity to emerge. Significant differences in the P1 time range were not found; however, this could be due to the long analysis window used (100 ms) or evaluation of recording sites that were not optimal for capturing P1 effects.

Given the differences in ERP quantification across studies [7,8], the observation of an enhanced P1 to dynamic PLWs appears to be in line with a number of previous studies. As described in the introduction, an enhanced P1 to dynamic PLWs was observed by Krakowski and colleagues [11] and early modulation of the MEG has been reported by other researchers [12,13]. Nonetheless, a study by Jokisch and colleagues [10] clearly shows no evidence of a P1 effect in response to intact PLWs. One possible explanation for this discrepancy is the fact that the present study, as well as those of others, used either entirely sideways-facing walkers [7,8,12,13], or a subset of sideways-facing walkers [11]. In contrast, the study by Jokisch and colleagues [10] employed only forward-facing walkers. It is possible that the sideways-facing walkers present a more salient and more readily processed depiction of the human form. Further research that directly compares the neurophysiological responses to forward-facing and sideways-facing walkers would be a welcomed addition to the present results.

## Conclusions

The current study provides evidence that the P1 ERP component is sensitive to both static and dynamic PLWs. The finding that the P1 was sensitive to static PLWs suggests that modulation of this component by dynamic PLWs can be attributed to configural processing alone. This finding has implications for dynamic form models of biological motion perception. These models posit that the percept of biological motion arises as a result of the temporal integration of the global form present in individual frames of PLW animations [6,32]. The rapid and reflexive processing of global form (postures), within approximately 100 ms of stimulus onset, supports the feasibility of such models, which presume action recognition based on sequences of static postures.
